# Case Report: A Novel Ventilated Thermoplastic Mesh Bandage for Post-operative Management of Large Soft Tissue Defects: A Case Series of Three Dogs Treated With Autologous Platelet Concentrates

**DOI:** 10.3389/fvets.2021.704567

**Published:** 2021-09-01

**Authors:** Priscilla Berni, Fabio Leonardi, Virna Conti, Roberto Ramoni, Stefano Grolli, Giovanni Mattioli

**Affiliations:** Department of Veterinary Science, University of Parma, Parma, Italy

**Keywords:** platelet-rich plasma, PRP, regenerative medicine therapy, large cutaneous wound, dog

## Abstract

A ventilated thermoplastic mesh bandage was used for the post-operative management of large soft tissue defects in three dogs. Once the granulation tissue appeared, the wounds were treated with liquid or jellified autologous platelet concentrates, Platelet Rich Plasma (PRP) and Platelet Lysate (PL), to improve the wound healing process. After cleaning the wound with sterile physiological solution, a dressing was performed with several layers of cotton. A window through the layers of cotton was opened above the wound. Then, the platelet concentrate was topically applied, and the bandage was completed by placing, over the access window, a ventilated thermoplastic mesh modeled according to the size and shape of the wound. After 24 h, it was replaced by a low adhesion bandage. The thermoplastic mesh avoids the direct contact between the wound and the external layers of the bandage, preventing the drainage of the topical agent and the removal of the growing healthy granulation tissue. The bandage proposed in this study is easily applied by the veterinarian and well-tolerated by the animal, ensuring high welfare standards in stressed patients presenting compromised clinical conditions.

## Introduction

Skin wounds are among the most common injuries in veterinary medicine. They include lacerations, abrasions, pressure injuries, punctures, incisions, burns, frostbite woundsand chemical burns (1). Wound healing and tissue repair usually occur following a sequence of four stages: haemostasis, inflammation, cell proliferation, and tissue remodeling (2). Wound healing is also related to the severity of tissue trauma, wound size and location, and the extent of microbiological contamination (3). Soft tissue wounds may heal either by first or by secondary intention. First intention healing refers to the direct apposition of wound edges (e.g., surgical wounds, recent traumatic injuries). Secondary intention healing refers to the healing of a wound where the edges cannot be approximated (e.g., extensive tissue loss, infected wounds); in this case, the reparative process is prolonged and occurs by contraction and re-epithelization (1, 4). Accurate wound assessment and its effective management are crucial aspects for selecting the most appropriate method of closure. It is crucial to prevent further wound contamination or infection by proper cleaning and irrigation, aiming to remove foreign debris and necrotic tissue (5). Secondary intention healing requires a granulation tissue formation which is an essential indicator of tissue viability. When secondary wound healing occurs, granulation tissue matrix fills the wound from the base, replacing necrotic tissue with new tissue and vasculature until scar tissue is wholly formed (6, 7). Wound healing by secondary intention can be facilitated by the topical application of antiseptics, antibiotics, cleansing agents, wound gel containing hyaluronic acid, and various natural products, including honey and sugar (1, 8, 9). In the last years, regenerative medicine approaches to promote the healing of extensive tissue loss, chronic wounds or non-healing wounds have been proposed. In particular, platelet concentrates and Mesenchymal Stromal Cells have demonstrated stimulatory and pro-regenerative effects following local application, as a consequence of the release of a large panel of growth factors and bioactive molecules (10–13).

Platelet concentrates refer to a group of autologous blood products used to improve tissue healing. Platelet Rich Plasma (PRP), the most widely used platelet concentrate, is a blood product with a supra-physiological number of platelets (i.e., at least 4 to 5-fold compared to untreated whole blood) concentrated in a small volume of plasma. When applied, either in liquid or gel form, it releases several platelet-related growth factors that promote resident stem cells activation, endothelial and epithelial regeneration, stimulate angiogenesis, haemostasis, collagen synthesis, and soft-tissue healing (14). Platelet Lysate (PL) is a cell-free supernatant rich in growth factors, released from the platelets after freeze-thawing of the PRP (15). PL is clinically used for skin regeneration and wound healing (16–18). A practical advantage of PL compared to PRP is that it can be stored frozen and used for consecutive applications (15), avoiding multiple stressful blood collections for the patient. The use of platelet concentrates in veterinary medicine is constantly increasing because it has shown promising experimental and clinical effects in wound healing, especially when a massive tissue loss or a chronic wound needs to be treated both in dogs and horses (19–25). Autologous platelet concentrates are commonly applied to cutaneous wounds as a gel or, alternatively, topically sprayed onto the wound surface or injected intra-lesionally (14, 26, 27). Following topical application, the wound is usually protected by a suitable bandage. When wet-to-dry or adherent dressings are used, the platelet concentrate can stick to the gauze layers, whose subsequent removal may detach healthy newly formed tissues from the surface of the wound, thus delaying healing. Taking into account this criticism, non-adherent (or low adhesion) dressings are most commonly applied with PRP gels to facilitate removal with minimal damage to the underlying tissue. Nevertheless, despite their name, non-adherent dressings may also adhere to portions of the wound surface and cause damages to the regenerating tissue (1, 28). The present work describes for the first time the use of a bandage for the postoperative management of large wounds in three dogs, where autologous concentrate has been combined to the application of a bandage with a ventilated, sterile thermoplastic mesh. This bandage does not adhere to the platelet matrix and allows the application of the product directly onto the wound, avoiding the removal of healthy granulation tissue and preventing the drainage of the topical agent used to promote and accelerate the tissue healing.

## Methods

### PRP and PL Preparation

PRP was prepared from autologous blood, following a standardized protocol based on two centrifugation steps (19–21, 29). Peripheral venous blood (40–60 ml) was collected into 20–60 ml syringes containing sodium citrate anticoagulant solution (50×; final concentration 0.38% v/v), and then transferred into 15 ml conical centrifuge tubes (ThermoFisher Scientific Nunc; Rochester, NY). Before processing, the blood was subjected to a complete hemogram and biochemical analysis. The preparation of the PRP was realized under a sterile hood, employing sterile labware and syringes. The blood was first centrifuged at 180 × g/20 min in a benchtop centrifuge equipped with a swinging rotor (AlC 4236A centrifuge, Italy), and the plasma containing the platelet fraction was collected by a sterile plastic pipette. The platelet cell pellet was separated from the platelet poor plasma (PPP) by a second centrifugation step at 900 × g/15 min. The platelet pellet was then resuspended in a small volume of PPP and cells were counted by a hematology analyser (Cell-Dyn 3500R, Abbott, Chicago, USA). PPP was added to the platelet suspension and a PRP with a platelet concentration of 10^9^/ml was finally obtained. An aliquot was immediately used for the first therapeutic application; when additional treatments were needed, the remaining PRP was aliquoted in 2 ml sterile Eppendorf tubes, stored at −80° and thawed for further applications as platelet lysate (PL). The aim of this procedure was to avoid multiple stressful blood collections for the patient. In one case (Case 3), platelet gels were prepared in sterile glass plates by the addition of 10% (v/v) sterile calcium gluconate solution 1,000 mg/10 ml (S.A.L.F., Italy) to PRP (for the first treatment), or PL (for the next treatments).

### PRP/PL Application and Bandage

**Figure 1:**
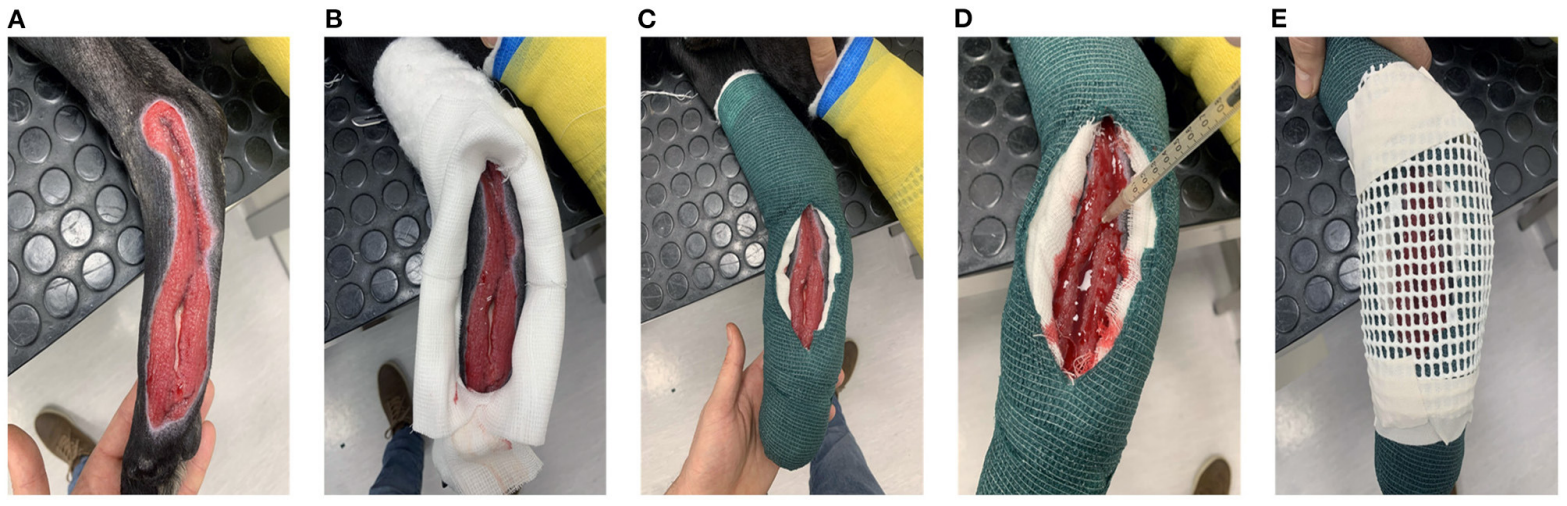
PRP application and bandage at the right tarsal and metatarsal regions in the hind limb of case 1. **(A)** Healthy and clean wound; **(B)** Window through the layers of cotton; **(C)** Dressing fixation with vetrap; **(D)** PRP application; **(E)** Thermoplastic mesh application.

**Figure 2:**
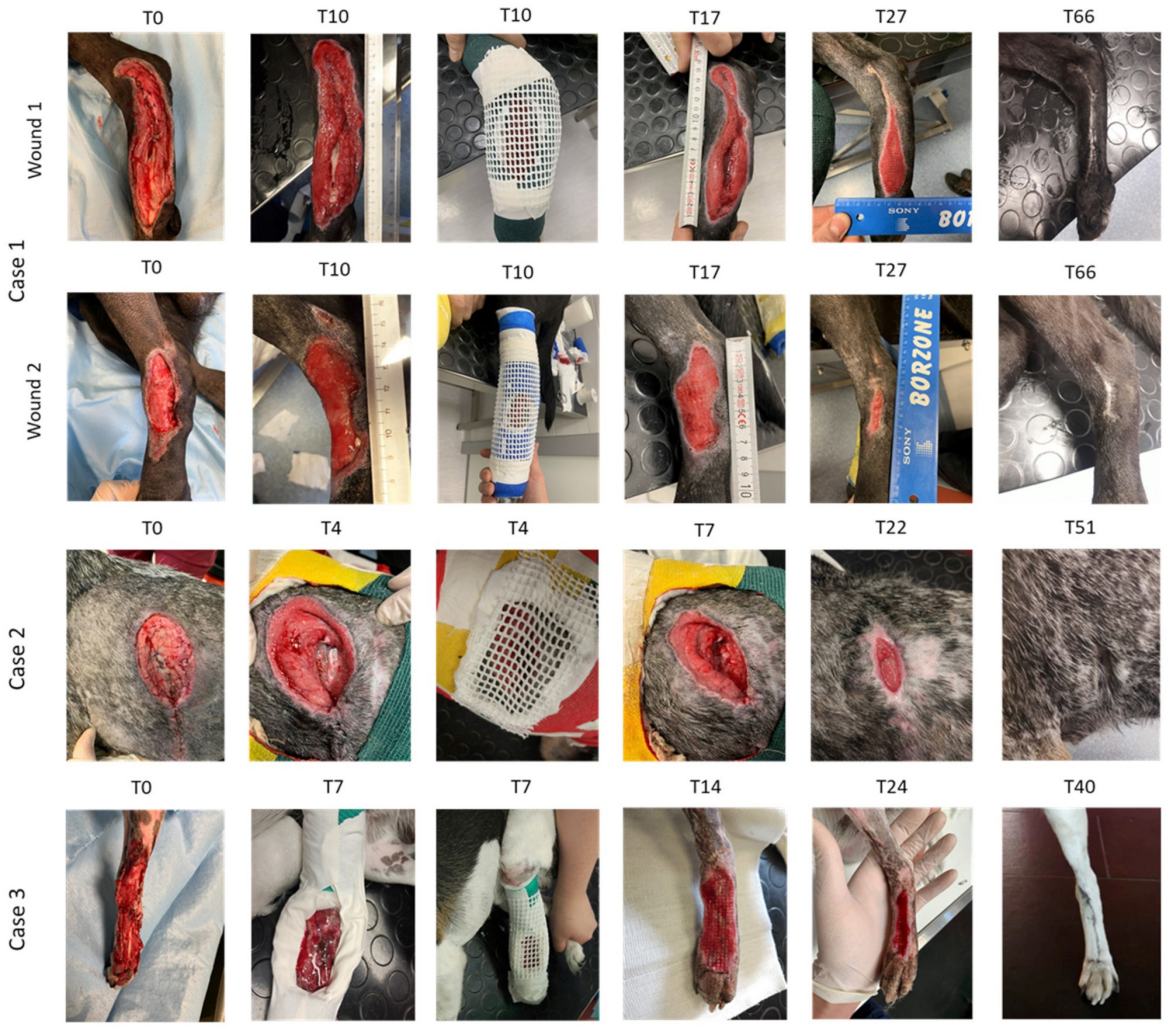
Serial macroscopic images of the wounds of the three cases at different time points. Wound 1, right limb. Wound 2, left limb.

PRP was applied when granulation tissue began to develop (Figures 1A, 2, case 1 at T0, case 2 at T4, case 3 at T7). In awake patients, a dressing was performed with several layers of cotton after cleaning the wound with abundant sterile physiological solution. A window through cotton layers was then opened above the wound (Figure 1B). Then, the dressing was fixed with a layer of cohesive elastic gauze bandage (vetrap) (Genia, Saint-Hilaire-de-Chaléons, France) (Figure 1C). A total of 3–6 ml (depending on the area of the wound) of PRP or PL was inoculated along the margin of the wound, between the skin and the granulation tissue (every 5 mm), and in the wound center (Figure 1D). Furthermore 1–2 ml of PRP or PL were dropped over the entire area of the defect. In Case 3, a PRP or PL-gel was layered onto the wound surface, in order to fill the entire thickness of the lesion. A ventilated thermoplastic mesh (Vet lite bandage, Kruuse, Langeskov, Denmark), modeled by heating according to the size of the wound, was placed to the window to avoid contact between the dressing and the wound surface (Figures 1E, 2: case 1 at T10, case 2 at T4, case 3 at T7). The plastic mesh was finally fixed with vetrap. This dressing was left *in situ* for 24 h, and then replaced by a low adherence dressing, formed by layers of gauze and cotton, covered, and fixed with vetrap. The bandage was changed every 2 days until complete wound healing. When additional treatments were needed, the windowed bandage with the thermoplastic mesh was applied again following the same procedure described above.

## Case Details

### History and Clinical Findings

Three client-owned dogs were referred to the Veterinary Teaching Hospital of the University of Parma for large soft tissue defects or chronic skin wounds. Animals details are shown in Table 1. All owners signed a written consent after having been notified of the therapeutic treatment. The dogs included in the study presented large defects in different regions of the body, due to different etiologies. For each patient, hematological and biochemical tests, wound skin swabs and radiologic examination were performed, whereas ultrasound examination was performed only for Case 2. During the period of hospitalization, antibiotic, anti-inflammatory, analgesic, symptomatic, and fluid therapies were performed as needed.

**Table 1:** Summary of cases included in this study regarding the age, weight, breed, and type and site of the wound.

**Case**	**Age (years)**	**Weight (kg)**	**Breed**	**Wound type**	**Wound site**
Case 1	6	30	Mixed breed	Two laceration wounds	Tarsal and metatarsal region of the hind limbs
Case 2	6	20	Griffon Bleu de Gascogne	Multiple wounds	Left chest
Case 3	3	9.9	Mixed breed	Degloving injury	Tarsal and metatarsal region of the left hind limb

#### Case 1

A 6-year-old intact male mixed breed dog, weighing 30 kg, was referred for two laceration wounds to the tarsal and metatarsal regions in the hind limbs, combined with bone exposure in the right limb (Figure 2, Case 1, wound 1 at T0 and wound 2 at T0). The etiology was unknown. The initial treatment (T1) was the surgical debridement of the two wounds. Before the appearance of granulation tissue, which is the prerequisite for the PRP/PL treatment, hyaluronic acid (Connettivina, Fidia Farmaceutici, Italy) was applied as an ointment twice a day; the wounds were covered with sterile gauzes, and soft bandages were applied. PRP and PL, both in liquid form, were applied at T10 and T17, respectively (Figure 2, Case 1). The patient was discharged from hospital after 12 days (T12). The postoperative care included antibiotic therapy for 7 days, and a wound control combined with dressing change every 2 days.

#### Case 2

An 8-year-old female Griffon Bleu de Gascogne, weighing 20 kg, was referred for multiple wounds to the left chest due to wild boar tusks. The wounds were combined with multiple rib fractures, and diaphragmatic and caudal pulmonary lobe lacerations. A pulmonary lobectomy was performed, and the diaphragmatic defect was closed. The patient was discharged after 9 days. Five days after hospital discharge, the patient was referred for the wound dehiscence in the left hemithorax (Figure 2, Case 2, T0). Due to intraoperative complications during the previous general anesthesia, instead of performing a new surgical procedure, the wound was treated with PRP gel at T4, when granulation tissue appeared, and with PL gel at T7, T12, and T22 (Figure 2, Case 2). The patient was discharged after 17 days (T17), and cage rest was prescribed. The dog was examined once a week until complete wound healing.

#### Case 3

A 3-year-old intact male mixed breed dog, weighing 9.9 kg, was referred for a car accident. The dog exhibited a degloving injury with bone exposure to the left tarsal and metatarsal dorsal region of the left hind limb (Figure 2, Case 3, T0). Initial treatment (T1) included surgical debridement. During the period of the hospitalization (3 days) and before the granulation tissue was formed, the wound was medicated with collagenase ointment (Iruxol, Smith&Nephew, London, UK) twice a day, covered with sterile gauzes, and a soft bandage was applied. PRP was applied as liquid form at T7 (Figure 2, Case 3, T7). The postoperative care included antibiotic therapy for 7 days, analgesic and anti-inflammatory therapy combined with gastroprotective drug for 5 days, and a visual examination of the wound combined with dressing change every 2 days for 8 days. Then, the dog was examined once a week until complete wound healing.

### Outcome and Follow-Up

After hospital discharge, the wounds were managed at home by the owner and Elizabethan collar was prescribed until complete wound healing. The owners were contacted daily by phone and asked about clinical evolution (e.g., loss of appetite, vocalization, mobility, response to touch, demeanor, posture/activity). The patient was examined once a week until complete wound healing. For Case 1, the wounds were examined once a week for 7 weeks until complete wound closure, achieved by day 66 (Figure 2). For Case 2, the wound was examined for 4 weeks until complete closure, achieved by day 51 (Figure 2). For Case 3, the wound was examined for 4 weeks until complete wound closure, achieved by day 40 (Figure 2). In Case 1 and Case 3, in which the wounds were located at the limbs, an orthopedic evaluation was performed to assess the Range of Motion (ROM) and the degree of lameness during the post-operative examination. In Case 1, no alteration of ROM nor lameness were found. In Case 3, the dog suffered from a tibio-tarsal subluxation caused by the car accident and presented a 3 out 5 grade lameness (moderately lame when walking) combined with joint instability. No wounds showed signs of infection, necrosis or other wound complications during the post-operative controls. Complete reepithelization and hair regrowth occurred in all three cases. Wound dehiscence no longer occurred.

## Discussion

In the present case series, a bandage associated with a ventilated white thermoplastic mesh is proposed to avoid direct contact between tissue treated with PRP and dressing cotton layers, optimizing the use of platelet concentrates. Cutaneous wound healing is a complex process, depending on various factors, including the size and severity of the trauma, position of the wound, tension and mobility of the margins and susceptibility to infection (30, 31). When large cutaneous lesions are present, healing occurs by second intention. Inadequate blood supply, bacterial contamination, tissue necrosis, inflammation, and hypertrophic scars are possible complications resulting in a delayed and poor healing (19). In these cases, a variety of therapeutic agents could be used to enhance wound healing. Hyaluronic acid and/or collagenase ointments are very important in the early treatment of full thickness wounds to maintain a moist environment and prevent or treat wound infection. Collagenase is a naturally occurring proteolytic enzyme that may be useful for debridement due to its ability to degrade collagen and elastin. Hyaluronic acid helps maintain a moist environment and is likely to play a role in the inflammation and granulation phases of healing (5–9). The use of PRP has been demonstrated that represents a good regenerative therapeutic strategy and favors the cutaneous wound healing process (19, 20). PRP promotes cell proliferation, differentiation, and neo-angiogenesis by releasing various autologous platelet-derived growth factors (14). In the present study, PRP was used when large cutaneous lesions occurred (case 1 and 3) and when the wound dehiscence occurred in a high-risk patient (case 2). In all cases, PRP treatment was initiated when the wound presented no evidence of infection, and a healthy granulation tissue started to develop. Granulation tissue is a contractile structure, histologically characterized by the presence and proliferation of fibroblasts, keratinocytes, endothelial cells, new thin-walled capillaries, and inflammatory cell infiltration of the extracellular matrix (2). PRP is characterized by a high concentration of activated blood platelets, which release pro- and anti-inflammatory mediators, cytokines and growth factors (GFs), including platelet-derived growth factor (PDGF), transforming growth factor-beta (TGF-β) and vascular endothelial growth factor (VEGF). Consequently, platelets significantly contribute to neo-angiogenesis, chemotaxis of neutrophils and macrophages, fibroplasia and epithelialization, promoting proper wound healing (25, 32, 33). PRP releases high levels of growth factors at very early time points, and significant secretion continues for a few days (34), so it is fundamental to let the PRP act for a proper time to fully exploit its biological potential. Various PRP application techniques have been described (local infiltration, spraying, association with 3D scaffolds) (19–26). In this study, the bandage associated with a ventilated thermoplastic mesh avoids direct contact between tissues treated with PRP and dressing cotton layers, optimizing the tissue absorption of the topical agent, while preventing its drainage by the bandage. Another critical point favoring wound healing is that the proposed bandage preserves the integrity of the new granulation tissue that, with conventional bandages where cotton gauze adheres on the surface of the wound, can be easily scrapped during the curettage. Other interesting features are the light weight, the possibility of modeling the right shape and size and translucence to X-rays. The veterinarian can easily apply this bandage and the patient immediately becomes confident with it, thus ensuring animal welfare during convalescence. The bandage is left *in situ* for 24 h and then replaced by a low adhesion bandage because prolonged wound exposure to air increases the risk of infection and prevents the maintenance of a moist environment (1). Nevertheless, as shown in case 1 and 2, it can be repeated when further PRP/PL treatments are required.

In conclusion, although limited by the small number of treated dogs and by the lack of controls, the outcomes of this study suggest that the proposed bandage provides the following practical advantages related to a fenestrated dressing: facilitates local wound care, relieves the pressure at the site, preserves the integrity of the healing tissue and ensures future bandage changes without the removal of the entire dressing. Furthermore, avoiding the direct contact between the thermoplastic mesh and the wound, this bandage allows a prolonged absorption of the topical agents used to enhance wound healing in companion animals.

## Data Availability Statement

The original contributions presented in the study are included in the article/supplementary material, further inquiries can be directed to the corresponding author/s.

## Ethics Statement

The present report did not use any animals for the purpose of scientific discovery. This is a retrospective study and does not require ethical review process by the Animal Welfare Committee of the University of Parma. The present retrospective clinical study was carried out with client owned dogs. All owners signed a written consent after having been discussed the properties of the treatment. Written informed consent was obtained from the owners for the participation of their animals in this study.

## Author Contributions

PB and FL: contributions to conception and design and acquisition of clinical data. GM and FL: contribution to diagnosis, surgical procedures, anesthetic management, and post-operative management. PB, VC, RR, and SG: contributions to laboratory test, PRP preparation, and application. All authors contributed equally to the writing of the manuscript, read, and approved the final manuscript.

## Conflict of Interest

The authors declare that the research was conducted in the absence of any commercial or financial relationships that could be construed as a potential conflict of interest.

## Publisher's Note

All claims expressed in this article are solely those of the authors and do not necessarily represent those of their affiliated organizations, or those of the publisher, the editors and the reviewers. Any product that may be evaluated in this article, or claim that may be made by its manufacturer, is not guaranteed or endorsed by the publisher.
